# Physician assistants in outpatient medical practice in Germany – opportunities and challenges

**DOI:** 10.3205/000351

**Published:** 2025-11-17

**Authors:** Stefan Bigge

**Affiliations:** 1 Tønder, Denmark

## Abstract

The German healthcare system faces considerable challenges, particularly due to a shortage of physicians in rural areas and increasing demands from an aging and multimorbid population. Physician assistants (PAs) represent a promising solution to address these challenges. As academically trained healthcare professionals, they take on delegable tasks such as medical histories, routine examinations, documentation, and the care of chronically ill patients. This relieves physicians and allows for more intensive patient care.

This paper examines the advantages of integrating physician assistants (PAs) into outpatient care. It shows that PAs can significantly improve the efficiency and quality of care through their work. In particular, PAs play a crucial role in disease management programs (DMPs) for the long-term care of chronically ill patients. Their involvement leads to a reduction in complications and an increase in patient satisfaction. From an economic perspective, PAs optimize the use of resources and help stabilize practices, especially in underserved regions.

Despite these potentials, there are significant challenges. There is a lack of uniform national legal regulations clearly defining the competencies and fields of activity of PAs. In addition, there are uncertainties regarding the refinancing of PA services, as well as issues of acceptance among physicians and patients. Competition with existing healthcare professions such as medical assistants (MFAs) or specialized roles like VERAHs necessitates clear delineation of responsibilities.

This paper concludes by presenting strategies for the successful integration of physician assistants (PAs). In addition to creating clear legal frameworks and refinancing models, promoting interdisciplinary collaboration is essential. Regular continuing education and the use of digital technologies such as telemedicine can further expand the competencies of PAs. The findings underline that PAs can play a crucial role in ensuring sustainable and patient-centered healthcare – especially in regions affected by physician shortages.

## Abstract (de)

Das deutsche Gesundheitssystem steht vor erheblichen Herausforderungen, insbesondere aufgrund eines Ärzt:innenmangels in ländlichen Regionen sowie steigender Anforderungen durch eine alternde und multimorbide Bevölkerung. Physician Assistants (PAs) stellen eine vielversprechende Lösung zur Bewältigung dieser Herausforderungen dar. Als akademisch ausgebildete Gesundheitsfachpersonen übernehmen sie delegierbare Aufgaben wie Anamnese, Routineuntersuchungen, Dokumentation und die Betreuung chronisch kranker Patient:innen. Dies entlastet Ärzt:innen und ermöglicht eine intensivere Patient:innenversorgung.

Dieser Beitrag untersucht die Vorteile der Integration von Physician Assistants (PAs) in die ambulante Versorgung. Er zeigt, dass PAs durch ihre Tätigkeit die Effizienz und die Versorgungsqualität deutlich verbessern können. Insbesondere in Disease-Management-Programmen (DMP) für die Langzeitbetreuung chronisch erkrankter Patient:innen spielen PAs eine zentrale Rolle. Ihre Einbindung führt zu einer Reduktion von Komplikationen und zu einer höheren Patient:innenzufriedenheit. Aus ökonomischer Perspektive optimieren PAs den Ressourceneinsatz und tragen zur Stabilisierung von Praxen bei, vor allem in unterversorgten Regionen.

Trotz dieser Potenziale bestehen erhebliche Herausforderungen. Es mangelt an einheitlichen nationalen rechtlichen Regelungen, die Kompetenzen und Tätigkeitsfelder von PAs klar definieren. Zudem bestehen Unsicherheiten hinsichtlich der Refinanzierung von PA-Leistungen sowie Fragen der Akzeptanz seitens Ärzt:innen und Patient:innen. Die Konkurrenz zu bestehenden Gesundheitsberufen wie Medizinischen Fachangestellten (MFA) oder spezialisierten Rollen wie VERAH erfordert eine klare Abgrenzung der Verantwortlichkeiten.

Der Beitrag schließt mit der Darstellung von Strategien für die erfolgreiche Integration von Physician Assistants (PAs). Neben der Schaffung klarer rechtlicher Rahmenbedingungen und Refinanzierungsmodelle ist die Förderung der interdisziplinären Zusammenarbeit essenziell. Regelmäßige Fortbildungen und der Einsatz digitaler Technologien wie Telemedizin können die Kompetenzen von PAs zusätzlich erweitern. Die Ergebnisse unterstreichen, dass PAs eine entscheidende Rolle bei der Sicherstellung einer nachhaltigen und patientenzentrierten Gesundheitsversorgung spielen können, insbesondere in Regionen, die vom Ärzt:innenmangel betroffen sind.

## 1 Introduction

The German healthcare system is facing increasing challenges. The shortage of physicians, especially in rural areas, along with the steadily growing demands on patient care due to an aging and multimorbid population, places considerable strain on healthcare structures [1]. The profession of physician assistant (PA), which originated in the USA and has been established there since the 1960s, has gained increasing significance in Germany since the mid-2010s [2]. These academically trained healthcare professionals are able to relieve physicians by taking on delegable tasks, thereby improving both the efficiency and quality of patient care [3].

The integration of physician assistants (PAs) into outpatient care is considered a promising measure to address the physician shortage and the overload of medical personnel. By taking on routine activities, PAs can not only relieve physicians but also improve the quality of care – for example, by reducing waiting times and enabling more intensive patient care [4], [5]. At the same time, legal, organizational, and economic obstacles hinder wider implementation and need to be overcome [1].

This publication investigates the benefits and challenges of integrating physician assistants (PAs) into outpatient care, with a special focus on general practices. The goal is to generate evidence-based insights into the contribution of PAs to improving quality of care and reducing the burden on physicians. In addition, necessary legal and organizational adjustments are identified in order to establish the framework conditions for sustainable integration of this professional group.

## 2 Background and context

The profession of physician assistant (PA) emerged in the United States in the 1960s as a response to the shortage of general practitioners. Since then, the profession has become internationally established and is increasingly being adapted in countries such as the Netherlands and Germany [2]. In Germany, training for PAs was first introduced in 2005 and has since evolved continuously. Nevertheless, the role of PAs in Germany remains less established and autonomous than in other countries, primarily due to differences in education and legal frameworks [3].

### Historical development and international role models

In the United States, physician assistants (PAs) have taken on a central role in the healthcare system. They work within a delegated model that allows them to perform a variety of tasks, such as conducting examinations and treating patients under the supervision of a physician [6]. This approach has proven effective in mitigating physician shortages [7]. In the Netherlands, a similar model has been implemented, with a strong emphasis on close interprofessional collaboration between physicians and PAs. In this context, the acceptance of PAs by the medical community has played a key role in their successful integration [8].

### The PA role in Germany

The training of physician assistants (PAs) in Germany differs significantly from that in the USA. While in the United States a master’s degree with extensive clinical and theoretical education is required, the German program is more practice-oriented and vocational. This puts greater emphasis on supportive tasks, while more complex responsibilities largely remain with physicians [9], [10].

In Germany, PAs primarily perform delegated tasks in outpatient care. A key area of their work is conducting long-term ECGs and blood pressure monitoring. These examinations are essential for diagnosing and monitoring cardiovascular diseases. PAs handle not only the technical implementation but also patient preparation and initial evaluations. Final diagnoses and treatment decisions remain the responsibility of the supervising physician [1].

A comparable situation exists with ultrasound examinations, which are part of routine diagnostics in many general practices. PAs can manage the entire process, from patient preparation to execution. This relieves physicians significantly, allowing them to focus on interpretation without spending time on the technical aspects of the procedure [11].

Another major area of responsibility for PAs is their involvement in Disease Management Programs (DMPs), which are particularly designed for chronic illnesses such as diabetes mellitus, asthma, and COPD. In these programs, PAs conduct regular check-ups, assess therapeutic goals, and adjust treatment plans following physician consultation. They also advise patients extensively on lifestyle changes, medication management, and treatment adherence. Their consistent and structured involvement enables long-term stabilization of chronically ill patients and helps reduce complications [12].

### Care challenges in outpatient practice

The shortage of physicians in Germany, particularly in rural areas, poses a significant challenge. According to the Federal Statistical Office, nearly one-third of practicing physicians are aged 55 or older, underscoring the urgent need for generational renewal [13]. At the same time, the age distribution of the population indicates a growing demand for medical care due to an increasingly elderly and multimorbid population. Rural regions are especially affected, where access to general medical care is becoming more difficult. In 2023, the average waiting time for an appointment in general practices in underserved regions was up to four weeks [13].

In this context, physician assistants (PAs) could play a key role in bridging care gaps. They can take over routine tasks, increase efficiency, and improve patient satisfaction by reducing waiting times [1]. Furthermore, many practices are burdened by staff shortages, which can negatively impact the quality of care. The support provided by PAs could relieve physicians, allowing them to focus more on complex cases and individual patient needs [12].

## 3 Type and methodology of the literature review

The literature review was conducted in a systematic and targeted manner to identify relevant scientific publications on the use of physician assistants (PAs) or similar medically assisting professions in outpatient care in Germany, as well as in international comparison.

### Objective of the review

The goal was to assess the current state of evidence regarding the role, effectiveness, and acceptance of PAs in outpatient care, along with existing challenges and structural conditions within the German healthcare system.

### Search strategy

The literature search was conducted between February and July 2025 using the following academic databases:

- **PubMed**
- **Web of Science**
- **Scopus**
- **Google Scholar** (to include gray literature)
- **SpringerLink** (for German-language journals such as *Gesundheitswesen* or *MMW*)

In addition, relevant websites such as **Destatis**, the **German Medical Association (BÄK)**, the **National Association of Statutory Health Insurance Physicians (KBV)**, and professional associations were reviewed.

**Search terms (examples, combined using Boolean operators):**

- “physician assistant” OR “Arztassistent” OR “PA”
- “ambulatory care” OR “primary care” OR “outpatient care”
- “Germany”
- “delegation” OR “substitution” OR “task sharing”
- “team-based care” AND “health workforce”
- “nurse practitioner” (for comparison and differentiation)

### Inclusion and exclusion criteria

**Inclusion criteria:**

- Peer-reviewed articles and studies (2010–2024)
- Publications focused on PAs or comparable professions in outpatient settings
- German- and English-language sources
- Empirical studies, reviews, overview articles, and position papers

**Exclusion criteria:**

- Publications solely focused on inpatient care without relevance to outpatient settings
- Purely theoretical articles without practical implementation relevance
- Duplicates and articles with unclear authorship

### Selection and analysis

The initial search yielded 93 results. After title and abstract screening, 36 publications were selected for full-text review. Ultimately, 14 articles were included in the final analysis based on their relevance, quality, and thematic fit. The selection process followed PRISMA principles, though a full systematic review was not intended.

The literature was categorized into the following thematic areas:

- Legal and structural framework conditions in Germany
- Job profiles and areas of deployment of PAs in outpatient care
- Acceptance by medical personnel and patients
- International comparisons regarding effectiveness and quality
- Economic impact and health policy implications

## 4 Advantages of integrating PAs

### Relief for medical staff

The integration of physician assistants (PAs) into outpatient care can provide significant relief for physicians, particularly in general practices. By taking on delegable tasks such as patient history, routine examinations, and documentation, PAs can significantly reduce physicians’ workloads [4]. This enables physicians to concentrate on more complex diagnostic and therapeutic cases that require their specialized expertise [5]. In emergency departments and general practices, the integration of PAs has led to optimized process organization and significant reductions in waiting times. This relief has a positive impact on physician job satisfaction by reducing administrative and operational pressures [6].

### Improved patient care

Another major benefit of integrating PAs is the improvement of patient care. Interprofessional collaboration between physicians and PAs ensures more intensive patient support. Studies show that such collaboration improves both the quality of care and patient satisfaction [14]. A key factor is the PAs’ ability to carry out regular check-ups and patient counseling within the framework of disease management programs (DMPs) for chronic conditions such as diabetes or COPD. This not only enhances continuity of care but also reduces complications and hospitalizations [2]. At the same time, delegating routine tasks allows for more intensive physician-patient interaction in complex medical situations [3].

### Economic benefits

Integrating physician assistants (PAs) into outpatient care offers significant economic advantages. Studies confirm that PAs can increase practice efficiency by taking on routine tasks that do not necessarily require a physician [6]. In countries like the USA and the Netherlands, the integration of PAs has led to reduced personnel costs without compromising the quality of care. In Germany, broader implementation of PAs could also contribute to the financial stabilization of practices, especially in regions affected by physician shortages and generational transitions [4].

### Continuing education and quality assurance

Ongoing training for physician assistants (PAs) is a crucial part of their professional development and is essential to ensure high-quality patient care. Only through regular training can PAs remain up to date with current medical research and technology and integrate new diagnostic and therapeutic methods into practice [3]. The implementation of structured continuing education programs is vital for the long-term integration of PAs into interdisciplinary teams. These programs should be practice-oriented and tailored to the specific demands of areas such as DMPs, telemedicine, and care for multimorbid patients [2]. Furthermore, continuous education contributes to increased patient safety. Regular training in error prevention, risk management, and evidence-based guidelines reduces treatment risks and sustainably improves care quality [6].

## 5 Challenges and difficulties

### Legal and organizational uncertainty

A major barrier to the integration of physician assistants (PAs) is the lack of uniform national regulations. Both the areas of responsibility and the competencies of PAs are currently not clearly defined. Their work is based solely on physician delegation, which leads to uncertainties – especially regarding the legal responsibility and liability of the delegating physicians [3]. This lack of clarity not only complicates the integration of PAs into medical teams but also limits their potential to take on responsibilities independently in patient care. A standardized legal framework could help overcome these challenges and strengthen the role of PAs in the long term.

### Acceptance among physicians and patients

Although physician assistants (PAs) offer added value to the healthcare system, there are reservations within the medical profession. PAs are often perceived as competitors who may diminish physicians’ responsibilities or question their professional status [4]. These perceptions often stem from unclear role definitions and insufficient communication. Simultaneously, patients often lack information about the PA profession. This information gap can lead to insecurity or mistrust. To promote acceptance, targeted education and transparent communication about the role and competencies of PAs are necessary [6].

### Cost factor and reimbursement

The training and integration of physician assistants (PAs) requires initial investment by medical practices. In addition, the reimbursement of certain services through statutory health insurance funds is currently insufficiently regulated, which reduces the economic appeal of employing PAs [14]. This unclear refinancing structure poses a particular challenge for practices in underserved regions. Including delegable tasks performed by PAs in the benefit catalog of statutory health insurance could increase the economic attractiveness of hiring PAs. Furthermore, funding programs could support the deployment of PAs in areas with a shortage of healthcare professionals.

### Competition with existing professional roles

The introduction of physician assistants (PAs) is sometimes perceived as competition to existing healthcare staff such as Medical Assistants (MFAs), nursing staff, or specialized assistants like VERAH or NäPa. Overlapping fields of activity can lead to tensions within teams and impair interprofessional collaboration [3]. To avoid such conflicts, clearly defined competencies and better role coordination are necessary. In addition, promoting interprofessional collaboration can help reduce tensions and increase efficiency in practice settings.

## 6 Strategies for the successful integration of PAs

### Interdisciplinary collaboration and team development

Acceptance of physician assistants (PAs) within the medical team is essential for their successful integration. Regular team meetings where the responsibilities and roles of PAs are transparently communicated form a central foundation for effective collaboration [4]. Such meetings not only clarify areas of responsibility but also promote knowledge sharing and the early resolution of potential conflicts.

A structured onboarding process is equally important. This should include targeted introduction to practice workflows and close supervision by experienced team members. A mentoring program could play a key role here, where experienced physicians guide new PAs, gradually expand their competencies, and familiarize them with interprofessional collaboration. These programs not only support the professional growth of PAs but also foster mutual trust among professional groups.

Moreover, integration can be strengthened through joint training and continuing education in which PAs, physicians, and other healthcare professionals participate equally. This enhances understanding of each other’s roles and strengthens team spirit. Another practical approach could be the definition of clear communication pathways – e.g., via digital tools that facilitate exchange between PAs and physicians and make workflows more transparent.

### Financing and economic viability

The establishment of transparent refinancing models is essential to make the employment of physician assistants (PAs) economically viable. Including delegable PA services – such as long-term ECGs or structured patient consultations – in the billing codes of statutory health insurance could make a significant contribution here [14]. In addition, funding programs could be introduced that specifically target rural and underserved regions to support the deployment of PAs. Such programs might include training subsidies or initial funding for practices hiring PAs for the first time.

### Development of continuing education and training programs

Ongoing education for physician assistants must be tailored to the specific demands of outpatient care. In addition to medical-technical skills, communication and organizational competencies should be promoted in order to enhance patient satisfaction and optimize interprofessional collaboration [6]. Additionally, regional continuing education days could be organized annually for all PAs. These events could disseminate up-to-date medical standards, legal updates, and best practices. They would support both individual skill development and create networks among PAs that facilitate knowledge exchange and joint problem-solving in the face of complex challenges [2].

### Legal and regulatory adaptations

The establishment of nationwide regulations is an indispensable prerequisite for the successful integration of physician assistants (PAs) into the German healthcare system. These regulations should clearly define the areas of activity for PAs, particularly regarding delegable medical tasks such as diagnostics, treatment monitoring, and patient counseling [3]. At the same time, legal responsibilities between PAs and supervising physicians must be clearly delineated to minimize liability issues.

Based on international models, expanded competencies – such as the prescription of certain medications under supervision – could be introduced in the long term to strengthen the autonomy of PAs [6].

## 7 Conclusion

Physician assistants (PAs) offer tremendous potential to make healthcare in Germany more efficient and of higher quality. By taking on delegable tasks, they relieve physicians and enable more intensive care for patients. In rural regions in particular, PAs can close care gaps and reduce the workload on medical staff.

However, significant challenges remain – such as the lack of nationwide regulations, unclear reimbursement models, and acceptance issues. A targeted adjustment of legal and organizational frameworks, as well as the promotion of interdisciplinary collaboration, is essential to ensure the sustainable integration of PAs.

Future developments could expand the competencies of PAs, for example through clearly defined autonomy in medical care. At the same time, digital technologies such as telemedicine open new possibilities for deployment, increasing the efficiency and reach of PAs. With these approaches, PAs can assume a key role in securing healthcare and contribute to a modern, patient-centered healthcare system in the long term.

## 8 Results

### Development of PA degree programs in Germany

In recent years, the academic training landscape for physician assistants (PAs) in Germany has expanded significantly. While only a handful of PA programs existed in 2018, by the winter semester 2024/25 there are 26 universities and colleges offering PA degrees, an increasing number of which are public institutions. The study offerings include both undergraduate bachelor’s degrees and advanced master’s programs, the latter of which have been available since 2021 [15].

Parallel to the growth in program availability, student enrollment has increased markedly: From approximately 260 first-year PA students in 2018, the number rose to more than 1,700 new enrollees in 2023/24. In total, over 5,000 students are currently enrolled in PA programs across Germany, representing a fivefold increase since 2019. Around 80% of students enrolled in PA programs are female [16], [17].

### Graduation rates and Master’s programs

Since the introduction of the PA degree in 2005, a total of 2,454 individuals have graduated with a PA bachelor’s degree in Germany (as of the 2023/24 academic year). The annual number of graduates continues to grow steadily, with 648 bachelor’s degrees conferred in 2022/23 alone. In addition, master’s programs are gradually being established. In 2023, 12 PA students completed a master’s degree, and four universities currently offer such postgraduate programs [15].

### Fields of employment

The majority of PA graduates currently work in inpatient hospital settings. An analysis of their distribution by medical specialty reveals the following key fields of employment:

- Surgery: The most common field of practice, including general, trauma, cardiovascular, and vascular surgery.
- Internal medicine: A major area of employment, particularly in cardiology, gastroenterology, and pulmonology.
- Emergency medicine: Many PAs support triage and acute care in emergency departments.
- Outpatient practice: Around 10% of PAs work in general or specialty outpatient practices, especially in the context of disease management programs or delegated diagnostic and counseling services.

Employers report a very high demand for PAs. Approximately 91% of graduates enter the PA workforce within three months of completing their studies, often in the same institutions where they completed their clinical placements [16].

### International comparison

Despite rapid domestic growth, Germany still lags behind leading countries in terms of PA workforce density and scope of practice:

- United States: Over 168,000 certified PAs (as of 2022), with tens of thousands of new graduates annually and broad clinical competencies.
- Netherlands: Approximately 2,000 practicing PAs in a population of 17.5 million, equivalent to 1.2 PAs per 10,000 inhabitants, compared to just 0.25 PAs per 10,000 inhabitants in Germany (2020).
- United Kingdom: Around 4,000 Physician Associates working within the National Health Service (NHS) (2023), with government support for expansion and increasing integration into hospital and general practice teams [18].

These international examples highlight the substantial potential of the PA profession and offer valuable guidance for further development and integration in the German healthcare system.

## 9 Discussion

The results of this article confirm the dynamic growth and increasing relevance of the physician assistant (PA) profession in Germany. While still relatively new, PA education and deployment have expanded rapidly. More than 5,000 students are currently enrolled in PA programs, and over 2,400 individuals have graduated as of 2024. Nevertheless, the role of PAs in outpatient care remains underdeveloped, particularly due to legal and financial constraints.

PAs in Germany primarily work in inpatient hospital settings, with surgery, internal medicine, and emergency medicine being the most common specialties. Their deployment in outpatient practices, especially in general medicine, remains limited. This aligns with findings from earlier surveys indicating that the lack of refinancing mechanisms and unclear delegation boundaries hinder wider PA utilization in ambulatory settings. While some practices are willing to delegate responsibilities to PAs, the absence of reimbursement options poses a significant barrier.

Legally, the PA profession in Germany lacks a dedicated regulatory framework. PAs operate exclusively under delegated authority without autonomous decision-making rights. In contrast to other healthcare professions, there is no federal law regulating PA training, scope of practice, or professional licensure. This creates uncertainty among employers and limits the degree to which PAs can assume responsibility in care delivery. Consequently, many PAs remain confined to supportive roles with limited direct patient management responsibilities.

Nonetheless, acceptance among medical teams appears to be increasing. Surveys report high satisfaction among PAs and generally positive interprofessional collaboration. However, broader public and physician awareness remains limited, and patient skepticism persists, particularly among older individuals and those unfamiliar with the profession. Overall, while acceptance is growing, it is still in a trial phase.

International comparisons offer valuable insights. In the United States, PAs are well-integrated across all healthcare sectors and are authorized to diagnose, treat, and prescribe medications under collaborative agreements. With more than 168,000 certified PAs, the profession is widely accepted and supported by national legislation. The Netherlands similarly grants PAs broad autonomy, including prescribing rights and defined independent procedures, following national registration under the BIG Act. Dutch PAs are embedded in both inpatient and primary care settings, contributing to improved efficiency and continuity.

In contrast, the United Kingdom has made slower progress. PAs (“physician associates”) were unregulated until 2024 and lack prescribing rights. However, they are increasingly deployed in NHS primary care and hospital teams. Official regulation under the General Medical Council (GMC) is now underway, with plans to expand their role cautiously. This cautious regulatory evolution reflects ongoing debates around patient safety and role boundaries.

Taken together, these systems illustrate key conditions for successful PA integration: clear legal authority, national registration, reimbursement frameworks, and structured interprofessional collaboration. Germany currently lacks several of these components.

Based on the analysis, the following recommendations can be made:

- Establish a dedicated legal framework for PAs, including national licensure and defined scopes of practice. Germany should consider a PA law analogous to the Dutch model, ensuring professional recognition and enabling limited independent practice.
- Develop reimbursement models for PA services in outpatient care. This could include billing codes for delegated services or incentive funds for practices employing PAs. Pilot programs should evaluate the cost-effectiveness of PA integration.
- Promote structured interprofessional team development. Defining PA roles clearly within care teams and fostering collaborative culture can improve acceptance. Joint training modules involving physicians and PAs can help reduce professional resistance.
- Launch public awareness and physician education initiatives. Clarifying the qualifications and competencies of PAs will increase trust among patients and support broader uptake.
- Expand postgraduate training and specialization pathways for PAs. Advanced clinical training programs and formal certification (e.g. DHPA exam) would enhance professional growth and retention.
- Implement continuous evaluation. Quality assurance through data collection on patient outcomes, efficiency, and satisfaction is essential to guide future adjustments.

In conclusion, physician assistants offer real potential to strengthen outpatient care in Germany, especially in underserved regions. Drawing from international experiences, Germany should take deliberate steps to address regulatory, financial, and cultural barriers. With a clear legal status, strategic funding, and ongoing evaluation, PAs can evolve into a core component of team-based, patient-centered care.

## Abbreviations

- COPD: Chronic obstructive pulmonary disease
- DMP: Disease management program
- ECG: Electrocardiogram
- MFA: Medical assistant
- NäPa: Non-physician practice assistant
- PA: Physician assistant
- VERAH: Care assistant in general practice

## Notes

### Competing interests

The author declares that he has no competing interests.
